# Using a Cloud Computing System to Reduce Door-to-Balloon Time in Acute ST-Elevation Myocardial Infarction Patients Transferred for Percutaneous Coronary Intervention

**DOI:** 10.1155/2017/2963172

**Published:** 2017-08-16

**Authors:** Chi-Kung Ho, Fu-Cheng Chen, Yung-Lung Chen, Hui-Ting Wang, Chien-Ho Lee, Wen-Jung Chung, Cheng-Jui Lin, Shu-Kai Hsueh, Shin-Chiang Hung, Kuan-Han Wu, Chu-Feng Liu, Chia-Te Kung, Cheng-I Cheng

**Affiliations:** ^1^Department of Public Health, Kaohsiung Medical University, Kaohsiung, Taiwan; ^2^Emergency Department, Kaohsiung Chang Gung Memorial Hospital, Kaohsiung, Taiwan; ^3^Chang Gung University College of Medicine, Kaohsiung, Taiwan; ^4^Division of Cardiology, Department of Internal Medicine, Kaohsiung Chang Gung Memorial Hospital, Kaohsiung, Taiwan

## Abstract

**Background:**

This study evaluated the impact on clinical outcomes using a cloud computing system to reduce percutaneous coronary intervention hospital door-to-balloon (DTB) time for ST segment elevation myocardial infarction (STEMI).

**Methods:**

A total of 369 patients before and after implementation of the transfer protocol were enrolled. Of these patients, 262 were transferred through protocol while the other 107 patients were transferred through the traditional referral process.

**Results:**

There were no significant differences in DTB time, pain to door of STEMI receiving center arrival time, and pain to balloon time between the two groups. Pain to electrocardiography time in patients with Killip I/II and catheterization laboratory to balloon time in patients with Killip III/IV were significantly reduced in transferred through protocol group compared to in traditional referral process group (both *p* < 0.05). There were also no remarkable differences in the complication rate and 30-day mortality between two groups. The multivariate analysis revealed that the independent predictors of 30-day mortality were elderly patients, advanced Killip score, and higher level of troponin-I.

**Conclusions:**

This study showed that patients transferred through our present protocol could reduce pain to electrocardiography and catheterization laboratory to balloon time in Killip I/II and III/IV patients separately. However, this study showed that using a cloud computing system in our present protocol did not reduce DTB time.

## 1. Introduction

Primary percutaneous coronary intervention (PCI) is the recommended method of reperfusion for patients with ST segment elevation myocardial infarction (STEMI), especially when performed within 12 hours of symptom onset [1]. Pivotal trials have established the clinical benefits of primary PCI as the preferred reperfusion strategy in patients presenting with STEMI, including cases that require transfer to a PCI-capable center [2]. Immediate transfer to a PCI-capable hospital for primary PCI for patients with STEMI who initially are transported to a non–PCI-capable hospital is recommended with first medical contact-to-device time system goal of 120 minutes or less [1]. Door-to-balloon (DTB) time is defined as time interval between percutaneous coronary intervention hospital arrival and the time of the first balloon inflation during PCI for STEMI. Established guidelines recommend that the DTB time should be less than 60 minutes for transfer-in STEMI patients.

Previous studies have highlighted the substantial delays in interhospital transfer that result in delayed reperfusion and that can be associated with worse patient outcomes [3, 4]. The initial diagnosis of STEMI is based on electrocardiography (ECG). However, before transferring STEMI patients to PCI center, the physicians at an emergency department of a local hospital or regional hospital without the capability of primary PCI have to fax the printed ECG to the tertiary medical center and then contact an interventional cardiologist by phone, which takes a considerable amount of time and may prolong door-in-door-out time in the first hospital. For this reason, we invented a cloud computing system for a STEMI transfer network to facilitate the emergent transfer of AMI patients and to reduce the interval of the door-in-door-out time. Unfortunately, there is a paucity of studies evaluating whether specific transfer protocols can shorten the DTB time at the transferred hospital. Furthermore, the impact of a DTB of less than 60 minutes on clinical outcome is unknown. The specific aim of this study is thus to evaluate the proportion of STEMI patients transferred via this specific protocol that achieve a DTB time of less than 60 minutes and determine the subsequent impact on clinical outcomes.

## 2. Methods

### 2.1. Study Design

This was a retrospective cohort study involving a quality monitoring database review as part of an initiative to improve DTB times for acute STEMI patients. Two cohorts before and after implementation of the transfer protocol using the cloud computing system were compared, with the former group consisting of patients transferred through the traditional referral process and the latter group of patients transferred through transfer protocol.

### 2.2. Study Setting and Population

**Figure 1:**
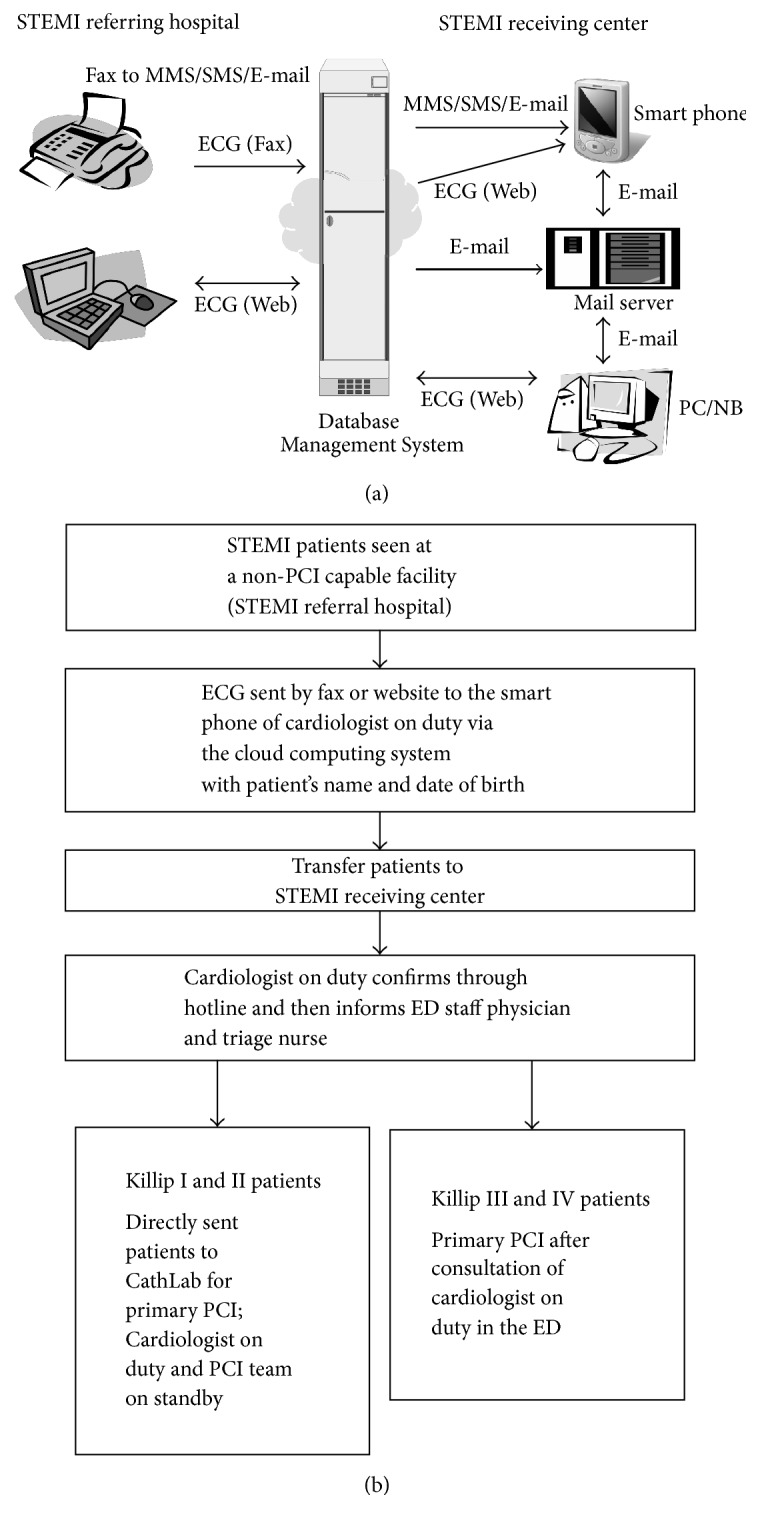
(a) Delivery of ECG information through cloud computing system. When a STEMI patient is seen at a non-PCI-capable facility hospital (STEMI referring hospital), the ECG of the patients is sent by a traditional fax machine or website to our cloud computing system, which automatically directs the ECG image to the smart phone of the cardiologist on duty at the STEMI receiving center. ECG: electrocardiography; MMS: multimedia messaging service; PCI: percutaneous coronary intervention; SMS: short message service; STEMI: ST segment elevation myocardial infarction. (b) STEMI transfer protocol. Once the cardiologist on duty confirms the diagnosis and calls back the referral physician to ascertain the medical condition of patients, he informs the ED staff physician as well as the triage nurse and immediately activates the PCI team in the STEMI receiving center. When the patients arrive at the ED of the STEMI receiving center, the patients with Killip I and II statuses are sent directly to the catheterization laboratory for primary PCI without the consultation of the cardiologist on duty. The patients who present as Killip III and IV statuses are stabilized at the ED and receive PCI after the consultation of cardiologist on duty. ED: emergency department; PCI: percutaneous coronary intervention; STEMI: ST segment elevation myocardial infarction.

Our institution is a 2000-bed tertiary care hospital located in Kaohsiung City in southern Taiwan. Primary PCI services and an active STEMI program running 24 hours and 7 days a week have been operational since 2001. Approximately 200 STEMI patients are treated per year, and one-third of them are referred from nearby and rural hospitals. We started to operate our STEMI transfer protocol via cloud computing system (Figure 1(a)) in October of 2012. When STEMI patients are seen at a non-PCI-capable facility hospital (STEMI referring hospital), the ECG of the patients is sent by a traditional fax machine or website to our cloud computing system which automatically directs the ECG image to the smartphone of the cardiologist on duty in the STEMI receiving center. Our STEMI transfer protocol is illustrated in Figure 1(b). Briefly, once the cardiologist on duty confirms the diagnosis from the STEMI ECG and calls back the referral physician to ascertain the medical condition of the patients via the hotline, he informs the emergency department (ED) staff physician as well as the triage nurse and immediately activates the PCI team in STEMI receiving center. When the STEMI patients are transferred from the STEMI referring hospital to the ED of the STEMI receiving center, the patients with Killip I and II statuses are sent directly to the catheterization laboratory for primary PCI without the consultation of the cardiologist on duty. The patients who present as Killip III and IV (advanced Killip score) statuses are stabilized at the ED and receive PCI after the consultation with the cardiologist on duty.

The inclusion criteria included patients aged 18 years or older who presented in the ED within 12 h of ischemic chest pain onset and who fulfilled the diagnostic criteria of acute STEMI by ECG (ST segment elevation > 1 mm in two contiguous limb leads and 2 mm in precordial leads or presence of new onset left bundle branch block) [5]. Exclusion criteria included patients who were considered unsuitable for PCI at the physician's discretion due to resuscitation for more than 30 minutes, refusal of primary PCI, having a pain to door of STEMI receiving center arrival time > 12 hours, or receiving fibrolytic treatment in referral hospital. Baseline characteristics, angiographic findings, and time interval difference, complication, and clinical outcome were obtained. Patient records and information were anonymized and deidentified prior to analysis. This study was approved by the institutional review board of Chang Gung Medical Foundation (102-4420B).

### 2.3. Data Analysis

Continuous data were presented as mean ± standard deviation and were analyzed using Student's *t*-test. Categorical data were presented as counts and percentages and were analyzed with the chi-square test. We calculated the median time and interquartile ranges for each time interval in minutes. Differences of each time interval between groups were assessed using the parametric Mann–Whitney *U* test. Pearson's test was used to assess the relation between troponin-I level and time intervals. SPSS for Windows (version 19.0; SPSS, Chicago, Ill, USA) was used for all the analyses. A two-tailed *p* value ≤ 0.05 was considered statistically significant.

## 3. Results

### 3.1. Patient Enrollment

There were 385 STEMI patients who were transferred and received primary PCI from January 1, 2011, to December 31, 2016. We enrolled 369 STEMI patients after excluding 16 patients (4 patients with prolonged cardiopulmonary resuscitation in the ED more than 30 minutes, 8 patients with pain to door of STEMI receiving center arrival time > 12 h, 2 patients with initial refusal of primary PCI, and 2 patients with fibrinolytic therapy in referral hospital). Finally, 262 patients were in the transferred through protocol group as opposed to 107 patients in the traditional referral process group.

### 3.2. Baseline Characteristics and Angiographic Findings

Table 1 lists the baseline clinical characteristics and angiographic findings of the transferred through protocol and traditional referral process groups. There were no significant differences between the two groups in terms of age, gender, hypertension, body mass index, previous myocardial infarction (MI) history, acute MI location, Killip score, troponin-I, systolic and diastolic blood pressures, and heart rate. The percentage of current smoking, diabetes mellitus, and hyperlipidemia were significantly higher in the transferred through protocol group than in the traditional referral process group (all *p* < 0.05). Additionally, the percentage of multivessel coronary artery disease diagnosed by cardiac catheterization was significantly higher in the transferred through protocol group than in the traditional referral process group (65.6% versus 36.4%, *p* < 0.001). There were no significant differences between the two groups in the percentage of achieving postprocedural Thrombolysis In Myocardial Infarction- (TIMI-) 3 flow and stenting.

### 3.3. Time Intervals and Troponin-I Level

**Figure 2:**
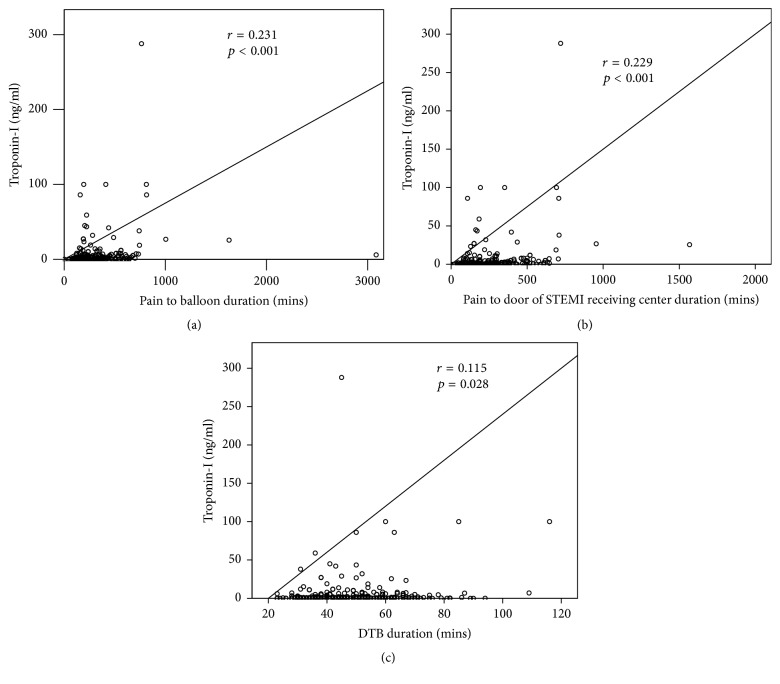
The correlation between troponin-I level and time intervals of pain to balloon, pain to door of STEMI receiving center arrival, and DTB. (a) The troponin-I level is significantly associated with time interval of pain to balloon (*r* = 0.231, *p* < 0.001). (b) The troponin-I level is significantly associated with time interval of pain to door of STEMI receiving center arrival (*r* = 0.229, *p* < 0.001). (c) The troponin-I level is not significantly associated with time interval of DTB (*r* = 0.115, *p* = 0.028). DTB: door-to-balloon time; STEMI: ST segment elevation myocardial infarction.

Between-group differences of time intervals are presented in Table 2. There were no significant differences between the two groups in door-to-ECG time, door-to-catheterization laboratory time, catheterization laboratory to balloon time, and DTB time. Besides, there were also no differences in the percentage of DTB time of less than 60 and 90 minutes between the two groups. There were also no differences between two groups in pain to door of STEMI receiving center arrival time, pain to ECG time, and pain to balloon time. However, pain to electrocardiography time in patients with Killip I/II and catheterization laboratory to balloon time in patients with Killip III/IV were significantly reduced in transferred through protocol group compared to in traditional referral process group (both *p* < 0.05). The correlation between pain to balloon time, pain to door of STEMI receiving center arrival time, and DTB time intervals and troponin-I level was shown in Figure 2. The level of troponin-I level was significantly associated with the time intervals of DTB, pain to door of STEMI receiving center arrival, and pain to balloon (all *p* < 0.05).

### 3.4. Clinical Outcomes and Complications

The complication rate and clinical outcomes of the patients are shown in Table 3. There were no significant differences between the two groups in patients receiving intubation, cardiopulmonary-cerebral resuscitation, intra-aortic balloon pump, and extracorporeal membrane oxygenation support. There were also no differences in the occurrence of ventricular tachycardia, ventricular fibrillation, or atrioventricular block, and no difference in the left ventricular ejection fraction between two groups. Finally, there were also no remarkable differences in the length of hospital stay and 30-day mortality.

Multivariate analysis showed patients with old age, advanced Killip score upon arrival, and higher level of troponin-I were independent predictors of 30-day mortality (Table 4).

## 4. Discussion

There are several important findings in this study. Firstly, DTB time was not significantly reduced with our present transfer protocol. However, pain to electrocardiography time in patients with Killip I/II and catheterization laboratory to balloon time in patients with Killip III/IV were significantly reduced with our present transfer protocol. Secondly, the complication rate and clinical outcomes were not further reduced using this present protocol. Finally, patients with old age, advanced Killip score upon arrival, and higher level of troponin-I had worse outcome than those without.

With great effort, the median DTB time decreased substantially and the percentage of patients whose DTB time was within 90 minutes increased from 44.2% to 91.4% over the 6 years ending in mid-2010s in the United States [6]. Several specific hospital strategies can account for this significantly reduced DTB time [7]. However, factors delaying time to reperfusion in primary PCI have been identified but vary according to countries, populations, and facilities of the STEMI networks involved [8]. For hospitals without a catheterization laboratory, it is imperative to establish partnerships with a STEMI receiving center. Once two institutions reach an agreement, the next step is to establish a simple STEMI transfer protocol for both hospitals to manage patients. Our cloud computing system can deliver the ECG fax or send it by website from STEMI referring hospital to the smart phone of the on duty doctor in the STEMI receiving center through a multimedia messaging service. The reception of the message is therefore not limited by time and space, and the doctor on duty can activate the primary PCI team after confirming the diagnosis of STEMI. This system can also transfer information through a short message service and e-mail, which can simplify and accelerate the transfer of the message. With this protocol, the pain to electrocardiography time was significantly reduced in patients with relatively stable hemodynamic status (Killip I/II) mainly from the shortened door-in-door-out time due to the transfer of the message being simplified and accelerated. In patients with Killip III/IV status, the referring hospital and ED of STEMI receiving center needed to stabilize the patients firstly and then consulted the cardiologist on duty. The cardiologist on duty can prepare for the hemodynamic support (IABP and ECMO) earlier when they received the information from the referring hospital and thus reduced the catheterization laboratory to balloon time. Furthermore, patients in the transferred through protocol group did not have a significantly lower mortality rate compared to those in the traditional referral process group. Patients who were transferred through our present protocol had more cardiovascular risk and multiple vessel disease, which was diagnosed when presented as acute STEMI. This may be due to the enrollment of the hospitals from rural areas in this present protocol. There are urban-rural differences in cardiovascular risk factors and patients may have fewer healthcare resources and lower educational status in rural areas [9–11]. The present study is also consistent with previous studies that elderly patients, advanced Killip score upon arrival, and higher level of troponin-I were remarkable predictors of 30-day mortality [12–15]. Our study also showed that the troponin-I level was significantly correlated to the time intervals of DTB, pain to door of STEMI receiving center arrival, and pain to balloon. According to previous and present studies, the troponin-I level was independently associated with clinical outcomes [16, 17]. Thus, no significant differences in DTB, pain to door of STEMI receiving center arrival time, and pain to balloon time between the two groups may account for the lack of difference in clinical outcome [18, 19]. Chen et al. also addressed the importance of reducing pain to door of STEMI receiving center arrival time and pain to balloon time in improving the clinical outcomes of those STEMI patients [19]. In that case, the promotion of public health education by teaching those rural citizens to be vigilant to symptoms of acute coronary syndrome may reduce the time of pain to door of STEMI receiving center arrival and pain to balloon and further improve the clinical outcomes of those STEMI patients transferred from rural areas.

## 5. Limitations

Our study has several limitations. First of all, this is a retrospective study analyzing a prospective enrollment. The differences in the baseline characteristics and angiographic findings between two groups may influence the analysis of the real impact of this present protocol on clinical outcome. However, patients with transferred through protocol still had a comparable outcome with patients with traditional referral process even when they had an increased cardiovascular risk and multiple vessel disease. Secondly, although we did not have detailed information of (1) pain to STEMI referring hospital arrival time, (2) duration of STEMI referring hospital stay, and (3) the comparison of management in STEMI referring hospital between the two groups, pain to door of STEMI receiving center arrival time did not differ between the two groups. This may reduce the possible influence of these factors on baseline variances and clinical outcome between two groups. Finally, further reducing the pain to door of STEMI receiving center arrival time and pain to balloon time by the promotion of public health education may be crucial to improve the clinical outcome. Further clinical trial should be performed.

## 6. Conclusions

Our study shows that the novel cloud computing systems facilitating the communications between STEMI referring hospital and STEMI receiving center can reduce the pain to electrocardiography time in STEMI patient with Killip I/II status and also the catheterization laboratory to balloon time in those with advanced Killip status. However, this study showed that using a cloud computing system in our present protocol did not reduce DTB time.

## Figures and Tables

**Table 1:** Baseline clinical characteristics and angiographic findings.

Variables	Transferred through protocol group (*n* = 262)	Traditional referral process group (*n* =107)	*p* value
Age (yrs)	61.0 ± 13.1	60.8 ± 13.6	0.905
Male gender	83.2% (218)	80.4% (86)	0.517
Current smoking	62.2% (163)	49.5% (53)	0.025
Hypertension	56.9% (149)	67.3% (72)	0.079
Diabetes mellitus	40.8% (107)	27.1% (29)	0.017
Old myocardial infarction	3.8% (10)	4.7% (5)	0.706
Body mass index (kg/m^2^)	25.4 ± 3.5	25.6 ± 3.9	0.763
Dyslipidemia	69.8% (183)	48.6% (52)	<0.001
Troponin-I^*∗*^	3.54 ± 11.54	7.51 ± 31.27	0.204
Killip class			0.415
1	64.9% (170)	58.9% (63)	
2	13.0% (34)	19.6% (21)	
3	7.6% (20)	8.4% (9)	
4	14.5% (38)	13.1% (14)	
Systolic blood pressure^*∗*^	137.6 ± 27.6	140.2 ± 32.8	0.426
Diastolic blood pressure^*∗*^	83.1 ± 19.7	95.0 ± 89.8	0.176
Heart rate^*∗*^	78.7 ± 20.4	77.7 ± 18.7	0.675
MI location			1.000
Anterior wall MI	50.4% (132)	50.5% (54)	
Nonanterior wall MI	49.6% (130)	49.5% (53)	
Multivessel disease	65.6% (172)	36.4% (39)	<0.001
Postprocedural TIMI-3 flow	92.0% (241)	87.9% (94)	0.213
Stenting	95.9% (254)	93.5% (100)	0.124

Data are expressed as mean ± SD or % (*n*); LV: left ventricular; MI: myocardial infarct; TIMI: Thrombolysis In Myocardial Infarction; *∗* indicated all the data were measured upon presentation.

**Table 2:** Time interval difference between traditional referral and transfer with protocol.

Variables	Transferred through protocol group (*n* = 262)	Traditional referral process group (*n* = 107)	*p* value
Door-to-ECG time (mins)	2 (1, 4)	2 (0, 4)	0.618
Killip I/II	2 (1, 4)	2 (0, 4.75)	0.921
Killip III/IV	2 (1, 5)	1 (0, 2)	0.944
Door-to-CathLab time (mins)	27 (20, 33)	28 (21, 36)	0.466
Killip I/II	25 (19, 33)	28 (21, 36)	0.060
Killip III/IV	31 (25, 36)	27 (20, 37)	0.438
CathLab-to-balloon time (mins)	18 (14, 22)	18 (13, 23)	0.497
Killip I/II	17 (14, 21.75)	16.5 (13, 21)	0.729
Killip III/IV	18 (13, 22.25)	22 (16, 31)	0.041
Door-to-balloon time (mins)	45 (37, 55)	47 (38, 58)	0.388
Killip I/II	43 (36, 54)	46 (36.25, 57.75)	0.375
Killip III/IV	51.5 (40.75, 61)	50 (44, 64)	0.672
Pain to door of STEMI receiving center time (mins)	163 (116, 264)	174 (118, 275)	0.309
Killip I/II	154 (115, 240)	180.5 (113, 274.75)	0.154
Killip III/IV	185 (122.25, 314.25)	160 (118, 247)	0.777
Pain to ECG time (mins)	165 (119, 266)	174 (118, 274)	0.123
Killip I/II	157.5 (117.5, 239.25)	186 (119.5, 286.0)	0.043
Killip III/IV	185 (125, 330.5)	161 (127, 244)	0.777
Pain to balloon (mins)	199 (161, 306)	214 (165, 327)	0.159
Killip I/II	194.5 (159.25, 270)	215.5 (160.5, 327.75)	0.056
Killip III/IV	229 (172, 381.25)	212 (176, 327)	0.619
Door-to-balloon time < 60 mins	85.1% (223)	79.4% (85)	0.183
Killip I/II	88.2% (180)	81.0% (68)	0.104
Killip III/IV	74.1% (43)	73.9% (17)	0.983
Door-to-balloon time < 90 mins	99.2% (260)	99.1% (106)	0.868
Killip I/II	100% (204)	100% (84)	1.000
Killip III/IV	96.6% (56)	95.7% (22)	0.847

Data are expressed as median (25% percentile, 75% percentile) or % (*n*); CathLab: catheterization laboratory; ECG: electrocardiogram.

**Table 3:** Complications and clinical outcomes.

Variables	Transferred through protocol group (*n* = 262)	Traditional referral process group (*n* = 107)	*p* value
Length of hospital stay	7.9 ± 14.3	6.2 ± 6.6	0.234
Intubation	11.5% (30)	13.1% (14)	0.660
CPCR	6.5% (17)	2.8% (3)	0.208
IABP	19.1% (50)	14.0% (15)	0.293
ECMO	3.4% (9)	0.9% (1)	0.292
VT/VF	8.4% (22)	7.5% (8)	0.837
AV block	6.5% (17)	10.3% (11)	0.212
LVEF by echocardiography^*∗*^	55.8 ± 13.8	56.4 ± 16.3	0.733
30-day mortality	5.3 (14)	5.6% (6)	0.919

Data are expressed as mean ± SD or % (*n*); AV: atrioventricular; CPCR: cardiopulmonary-cerebral resuscitation; ECMO: extracorporeal membrane oxygenation; IABP: intra-aortic balloon pump; LV: left ventricular ejection fraction; VT: ventricular tachycardia; VF: ventricular fibrillation; *∗* indicated echocardiography performed on the second day of ST segment elevation myocardial infarct.

**Table 4:** Multiple stepwise logistic regression analysis of predictors for 30-day mortality.

Variables	Odds ratio	95% CI	*p* value
Age	1.098	1.044–1.155	<0.001
Advanced Killip score^*∗*^	13.117	3.906–44.052	<0.001
Troponin-I	1.345	1.042–1.737	0.023

CI: confidence interval; PCI: percutaneous coronary intervention; TIMI: Thrombolysis In Myocardial Infarction; *∗* indicated Killip score ≥ 3.
